# Clinicians and Older Adults’ Perceptions of the Utility of Patient-Generated Health Data in Caring for Older Adults: Exploratory Mixed Methods Study

**DOI:** 10.2196/29788

**Published:** 2021-11-05

**Authors:** Ben Kim, Peyman Ghasemi, Paul Stolee, Joon Lee

**Affiliations:** 1 School of Public Health and Health Systems University of Waterloo Waterloo, ON Canada; 2 Data Intelligence for Health Lab Cumming School of Medicine University of Calgary Calgary, AB Canada; 3 Biomedical Engineering Graduate Program University of Calgary Calgary, AB Canada; 4 Department of Community Health Sciences Cumming School of Medicine University of Calgary Calgary, AB Canada; 5 Department of Cardiac Sciences Cumming School of Medicine University of Calgary Calgary, AB Canada

**Keywords:** mobile health, mHealth, older adults, wearables, patient generated health data, chronic disease management, home care, self-care, activities of daily living, sleep

## Abstract

**Background:**

Many people are motivated to self-track their health and optimize their well-being through mobile health apps and wearable devices. The diversity and complexity of these systems have evolved over time, resulting in a large amount of data referred to as patient-generated health data (PGHD), which has recently emerged as a useful set of data elements in health care systems around the world. Despite the increased interest in PGHD, clinicians and older adults’ perceptions of PGHD are poorly understood. In particular, although some clinician barriers to using PGHD have been identified, such as concerns about data quality, ease of use, reliability, privacy, and regulatory issues, little is known from the perspectives of older adults.

**Objective:**

This study aims to explore the similarities and differences in the perceptions of older adults and clinicians with regard to how various types of PGHD can be used to care for older adults.

**Methods:**

A mixed methods study was conducted to explore clinicians and older adults’ perceptions of PGHD. Focus groups were conducted with older adults and health care providers from the Greater Toronto area and the Kitchener-Waterloo region. The participants were asked to discuss their perceptions of PGHD, including facilitators and barriers. A questionnaire aimed at exploring the perceived usefulness of a range of different PGHD was also embedded in the study design. Focus group interviews were transcribed for thematic analysis, whereas the questionnaire results were analyzed using descriptive statistics.

**Results:**

Of the 9 participants, 4 (44%) were clinicians (average age 38.3 years, SD 7 years), and 5 (56%) were older adults (average age 81.0 years, SD 9.1 years). Four main themes were identified from the focus group interviews: influence of PGHD on patient-provider trust, reliability of PGHD, meaningful use of PGHD and PGHD-based decision support systems, and perceived clinical benefits and intrusiveness of PGHD. The questionnaire results were significantly correlated with the frequency of PGHD mentioned in the focus group interviews (*r*=0.42; *P*=.03) and demonstrated that older adults and clinicians perceived blood glucose, step count, physical activity, sleep, blood pressure, and stress level as the most useful data for managing health and delivering high-quality care.

**Conclusions:**

This embedded mixed methods study generated several important findings about older adults and clinicians’ perceptions and perceived usefulness of a range of PGHD. Owing to the exploratory nature of this study, further research is needed to understand the concerns about data privacy, potential negative impact on the trust between older adults and clinicians, data quality and quantity, and usability of PGHD-related technologies for older adults.

## Introduction

### Background

A recent national survey reported that Canadians aged ≥55 years have the highest rate of self-tracking of health data at 62.9%, whereas 38.73% track the data digitally using mobile health (mHealth) apps, consumer wearable devices, and smart medical devices [1]. Many individuals are motivated to track their health data, including physical activity and sleep quality, and optimize their well-being [2]. The diversity and complexity of the collected data has evolved over time with the advancement of sensors. Self-tracking of health data began with a collection of simple measurements such as weight, step counts, hours slept, and exercise logs, and has now demonstrated successful tracking of qualitative and subjective assessments such as mood and emotion [2]. The added complexity of self-tracked health data demonstrates the level of motivation and interest of the general population and the desire to improve one’s health and well-being.

Self-tracking of health data results in a large amount of data, often referred to as patient-generated health data (PGHD). PGHD is defined as “data created, recorded, gathered, or inferred by or from patients or their designees to help address a health concern” [3]. The key characteristic of PGHD is that its management and sharing are directed by patients. Similar concepts about collecting data from patients in natural settings exist, such as patient-reported outcome measures (PROMs) and ecological momentary assessment (EMA). PROMs are standardized data collection methods that are initiated by health care providers with the aim of evaluating the effectiveness of care [4]. PGHD differs from PROMs in its use of consumer technologies and in that the collection and sharing are patient-directed. EMA is a research-driven data collection method that allows participants to report occurrences of phenomena of research interest, such as symptoms, behaviors, or cognitive processes [5]. As with PROMs, EMAs are not patient-driven, and their purpose is to provide data for research.

Patients, health care providers, researchers, private industry, and governments share a similar vision of future health care where PGHD plays an important and significant role [6-10]. In the United Kingdom, PGHD is envisioned as one of the foundations for improving the quality of care and decreasing health care costs under the *Personalised Health and Care 2020* policy [9]. The plan to integrate PGHD into health care practice has also been shared by the US government, where PGHD will provide a holistic and longitudinal view of the patient’s health [11]. Although PGHD and related health monitoring systems can help older adults age in place, the way such technologies are used for geriatric care can decrease their effectiveness and even cause confusion or intimidation for older adults [12-14]. Although the increased interest in using PGHD is evident from a strong commitment by governments, successful adoption and implementation of required health information systems hinge on buy-in from care providers and users.

Despite the increased interest in PGHD, little is known about the opinions of clinicians and patients on PGHD. Common barriers to the use of PGHD by clinicians include unfamiliarity with the data, insufficient expertise in interpreting the data, and concerns about data completeness, reliability, and relevance [15]. Furthermore, the lack of time for any task outside of routine clinical practice, technical challenges including incompatibility between PGHD and electronic medical record systems, and uncertainty around privacy regulations hamper clinicians’ willingness to adopt PGHD [16-18]. Although these factors hinder clinicians from using PGHD, little is known about the opinions of older patients and the common barriers to adopting PGHD. Understanding the factors associated with the use of PGHD by older adults can inform policy makers, health care providers, software developers, and other stakeholders about PGHD and provide useful guidance.

### Research Objective

This study aims to explore the similarities and differences in the perceptions of older adults and clinicians’ with regard to how various types of PGHD can be used to care for older adults. We compared their attitudes toward different types of PGHD. This study extends the current literature by investigating the opinions of older adults and health care providers on the key factors that facilitate or hinder the use of PGHD.

## Methods

### Study Design

An embedded mixed methods design was used with the one-phase QUAN (qual) approach to explore the study objective. To introduce the topic of PGHD to the participants and set the scope of the focus group, we presented a case study that described an older patient being asked to collect PGHD to manage multiple chronic conditions [19]. The quantitative data collection was nested within the overall research design and performed after reviewing the case study through a questionnaire that was developed specifically for this study. Focus group interviews were conducted immediately following the completion of the questionnaire to probe the perceived barriers and key factors in using PGHD.

Research ethics approval for this study was received from the University of Waterloo Office of Research Ethics (ORE #40803). All participants provided written informed consent.

### Procedures

The Data Rating Questionnaire (Multimedia Appendix 1) was administered to measure participants’ perceived usefulness of PGHD. Demographic information and information regarding previous experience with mHealth apps and wearable technologies that generate PGHD were also collected. Two semistructured focus group interviews were conducted at the University of Waterloo and at the conference room of a health care organization. A set of questions was prepared and used by the interviewer as a guide to probe the participants’ perceived factors that facilitate and hinder the use of PGHD (Multimedia Appendix 2). The discussions were audio-recorded for analysis.

### Recruitment

Convenience sampling and snowball recruitment strategy were used to recruit 5 older adults and 4 clinicians. They were recruited from the Greater Toronto Area and the Waterloo-Wellington region in Ontario, Canada. An invitation email was sent to local clinicians and a research support group comprising over 60 older adults. Recruitment started in October 2019, and focus group interviews were conducted in December 2019.

### Data Collection and Analysis

#### Case Study

The case study described a 77-year-old man newly diagnosed with congestive heart failure with pre-existing type 2 diabetes, hypertension, and hyperlipidemia (Multimedia Appendix 3). The case study highlights the new responsibility given to the patient to collect and monitor a plethora of PGHD, including weight, blood pressure, blood glucose level, dietary intake, and medication log, using a variety of digital tools and a traditional paper journal. Participants reviewed the case study and were encouraged to ask questions about the types of PGHD presented and the role of information technology in collecting PGHD. The case study was used as an anchor for the focus group as some participants were unfamiliar with the topic.

#### Data Rating Questionnaire

A 26-item, 5-point Likert scale questionnaire (Multimedia Appendix 1) was developed based on the outlined definition of PGHD from the office of the national coordinator for health information technology of the US government [20] and from literature review [21]. The questionnaire categorized PGHD types based on the mode of data collection as either passively collected or actively collected. Passively collected data were generated without user input and included step count, sleep quality, and location information. Actively collected data were manually captured by patients on demand. Participants were asked to rate the perceived usefulness of each PGHD type based on the case study.

#### Focus Group Interviews

Two 30-minute focus group interviews were conducted and audio-recorded. We interviewed 6 and 3 participants in the first and second sessions, respectively. The first group comprised 5 older adults and 1 physiotherapist, whereas the second group comprised 2 nurses and 1 family physician. The composition of each session was based on geographic and logistical convenience, and the division between clinicians and older adults was unintentional.

#### Analysis

Descriptive statistics were performed to analyze demographic information and previous experience with mHealth apps, wearable devices, collecting PGHD, and Data Rating Questionnaire results. There were some missing data as some participants did not provide answers, and they were excluded from all quantitative analyses.

Focus group interviews were transcribed and read in their entirety. A constant comparative analysis strategy was used to code and categorize them into themes [22]. This inductive approach involved an iterative cycle of comparing the data with existing codes and themes, providing the researchers with a sense of frequency of the theme. This approach allowed researchers to investigate other aspects of the themes, including their extensiveness, intensity, internal consistency, and perceived importance [22]. The number of times each PGHD concept was mentioned was tallied regardless of who mentioned them (eg, if one participant mentioned a particular concept three times, it was counted as 3). All quantitative analyses were performed using R Studio, and qualitative analyses were performed using NVivo 12 (QSR International).

## Results

### Participant Characteristics

Of the 9 participants, 4 (44%) identified themselves as clinicians, including 1 primary care physician, 2 registered nurses, and 1 registered physiotherapist. The mean age of the clinicians was 38.3 (SD 7) years, and 3 of them were women. The remaining 56% (5/9) of the participants identified themselves as health care users. The mean age of this group was 81.0 (SD 9.1) years, and 4 of the 5 older adults were women (Table 1).

**Table 1:** Participant characteristics (N=9).

Participants			Age (years)		Sex
**Older adults**					
	Participant 1	82		Female	
	Participant 2	78		Female	
	Participant 3	94		Male	
	Participant 4	78		Female	
	Participant 5	69		Female	
**Clinicians**					
	C1-physiotherapist	47		Female	
	C2-primary care physician	39		Female	
	C3-registered nurse	30		Male	
	C4-registered nurse or educator	37		Female	

### Participant Exposure to PGHD

Of the 4 clinicians asked about their previous use of mHealth apps, 3 (75%) reported having used them to track dietary intake and calories, to monitor weight changes, and to improve exercise and training. These 3 clinicians also used a wearable device. Wearable devices were used to monitor step counts, physical activity levels, exercise intensity, sleep quality, and heart rate. Of the 5 older adults, 3 (60%) used either an mHealth app or a wearable device to monitor step counts only despite understanding that their wearable device offered monitoring of other PGHD.

### Thematic Analysis

#### Theme 1: Influence of PGHD on Patient-Provider Trust

Older adults and clinicians had conflicting views on the impact of PGHD on patient compliance. Older adults felt that monitoring PGHD increased the transparency of their (lack of) engagement in healthy behaviors. Older adults understood that increased transparency encouraged and motivated compliance, although this was not explicitly stated.

Participants stated:

...he just sits in that chair and watching TV and he can say “Oh I walk” but you didn’t. from here to the washroom to the kitchen; that’s not enough.

Participant #1

...[clinicians will] see whether they have done this. And that goes for the exercise programs too and not just say it but follow through.

Participant #3

I think the device would help the clinician know when somebody is sneaking a candy bar or somebody says that they go for a walk everyday, but they really only go twice a week.

Clinician #1

Clinicians expressed concerns about the increased transparency via PGHD and how it could lead to noncompliance with the use of the system and selective disclosure of PGHD by patients. Clinicians also perceived that older adults were afraid of the negative impact noncompliance would have on the patient-provider relationship and, in turn, on the quality of care they received from their providers:

The biggest one I have seen as a doctor is the fact that you’ve not been following your diet or your exercise plan so I’m not going to show you because now you know.

Clinician #2

So, [patients] are like, okay I’m not going to, I’m just going to skip it this day, because having no data is better than showing that I wasn’t following directions or doing it properly.

Clinician #3

...the perception of, you know, how much they want to help me, because of things like, you know, well I can only help if you help yourself and then the perception of, well you don’t want to help yourself, so how could that impact that relationship with the provider.

Clinician #3

Not all older adults agreed with the suspected tendency toward selective disclosure of PGHD. Two older adults expressed that they were less likely to share PGHD when they were noncompliant and inclined to share only compliant information. However, one participant was comfortable sharing their PGHD regardless of compliance:

...if you’re underperforming, you’re a little more likely not to want to tell everything that you do

Participant #2

But if I walk every day in the good weather—not this weather—I want him to know about it and I wouldn’t tell him I did if I didn’t do it.

Participant #3

I would tell him. If I walk only 5000 or 6000 I will tell him too.

Participant #1

Older adults and clinicians generally agreed on the benefit that increased transparency arising from PGHD sharing has on the care they provide or receive. Ultimately, clinicians viewed noncompliance with PGHD collection as an issue they could help prevent by gaining buy-in from patients. Patients also raised the need for additional education, which might improve the understanding of the need for PGHD.

#### Theme 2: Reliability of PGHD

The clinicians recognized the issue of accuracy of PGHD from mHealth apps and wearable devices and understood that they might not be perfect. Despite the inaccuracies, the perceived clinical value outweighed the alternative of having no data. However, the clinicians’ concerns about the reliability of PGHD stemmed from the perceived lack of trust in the patients’ ability to capture or share the data reliably:

You have to assume that the patient is wearing it for the majority of the time.

Clinician #1

Not remembering to do it...I was told I was supposed to track this and I’ve forgotten so many times.

Clinician #3

Older adults and clinicians perceived that the lack of clinical knowledge by patients leads to a collection of irrelevant PGHD and decreases the usefulness of the information. In contrast, older adults viewed education on self-management as a key component in understanding the importance of PGHD:

I guess it depends on who is looking at the data and if the person entering it can also appreciate or have some clinical background, because then they can say, okay I’ll use it and I’ll enter it, because it has usefulness for my clinical provider.

Clinician #3

...they really there to teach him, make sure that he understands what—he needs to understand that he needs to take his blood pressure medication everyday and they need to monitor that and see whether it’s working.

Participant #4

Gaming mHealth apps and wearable devices used by patients to collect favorable data were viewed as a threat to the reliability of PGHD. Clinicians acknowledged that this issue was not unique to PGHD and that it could happen to any self-reported information:

And how accurate is the data when it comes, so like if you learn to game the system, you can choose to, you know...in the case of like blood sugars, you know, take it later on, so that way it looks like it’s a better reading than it actually is.

Clinician #3

Shake your hand as though you’re walking.

Clinician #2

They could be lying about writing down their values, right, or they could be lying about the weight that they measure at their home scale or whatever, right.

Clinician #4

Clinicians emphasized the threat to the reliability of PGHD through the manipulation of mHealth and wearable systems. This was because clinicians were aware of the advancement in sensor technology that enabled some previously actively collected PGHD to be passively collected, such as blood glucose levels. Passive data collection increased the trust clinicians put in the quality of the data as it prevented data manipulation by patients:

Like blood glucose right now, like right now it’s under actively sensed data...because I guess you would have to do like a finger prick and then we do reading and then enter it in, but now there is technology that exists where you, you know, you attach, and all you have to do is put the device.

Clinician #3

I mean after having worked with patients and now having parents that are dealing with chronic conditions themselves, I really hope that at some point a lot of that data collection is passive.

Clinician #2

Overall, the reliability and accuracy of PGHD were disproportionately perceived as an issue by clinicians compared with older adults. Clinicians also alluded to old age as a potential challenge as the older generation is not as fluent with mHealth, wearable technology, and other devices that collect PGHD.

#### Theme 3: Meaningful Use of PGHD and Decision Support Systems

The uncertainty around the meaningful use of PGHD was expressed by both older adults and clinicians. Older adults were reluctant to share their PGHD with their clinicians as they were uncertain of the use of PGHD by their clinicians and the skill levels of their clinicians to use them:

That’s the thing; check up are they really doing this?

Participant #4

...he is not going to absorb it any more than we would.

Participant #3

A large amount of data was viewed as a major hindrance to the use of PGHD by both older adults and clinicians. Older adults felt *overwhelmed* when trying to review and understand the data. Older adults felt discouraged from sharing the data as they perceived that reviewing PGHD was a time-consuming task and felt that clinicians would not have enough time:

And you want to know what’s important for you and I think people can do these things but you have to do it in little steps too. This is kind of overwhelming, the whole thing.

Participant #2

...the doctor is just simply too busy, he’ll never look at all this information that we’re talking about here. He won’t have the time.

Participant #4

However, clinicians did not express lack of skills as a barrier to PGHD use. Instead, clinicians reiterated the issue of the volume of PGHD and acknowledged the lack of time to review and discuss PGHD before or during consultations:

...as a provider, like I wouldn’t want to be the one going through like excel sheets of data.

Clinician #2

If I’m looking at all of the data that’s available across like 20 different measures, how long do I have for a consult even, or how long do I have allocated for a meeting for this patient.

Clinician #3

Despite the issues of information overload and lack of time, clinicians saw clinical value in collecting more PGHD. Clinicians envisioned that PGHD could provide additional information when investigating the effectiveness of treatments, such as newly prescribed medications or behavioral changes:

I would say if it wasn’t a technological or a financial cost constraint to have, at least the passive data stuff all included and made available to the clinician, because then you can correlate things like, all right well...you know, they had a blood pressure issue, right. What were they doing at the time, what was your physical activity at the time or did they get a good night’s sleep before, you may not see that directly, but having that data wouldn’t hurt.

Clinician #3

...from a clinician perspective, but when you asked about the clinician versus patients, I think it’d be nice to have all this data.

Clinician #2

Clinicians had an extensive view on decision support systems as an essential part of operationalizing PGHD in the clinical context. A decision support system was perceived as a tool that could highlight the most relevant information and reduce the time taken to interpret the data. It was also viewed as an early warning system for patients with deteriorating health:

From the provider perspective, how is the data presented to me, is it a whole set of charts and numbers I have to go find and find trends? Or is it, is there a dashboard that comes up that easily [find] trends for you, because then I can look at it, I’m going, oh okay, I see a positive trend, here’s what I can, it’s actionable like you said, I can do something with it and provide guidance. If it’s just a whole bunch numbers and I have to see well how close is it and how much time will that take, then I may be less, I may be more hesitant to ask for this data or use this data.

Clinician #3

...with maybe mental health issues or support issues, like depression, with their consent I think that would be great...if suddenly their social media usage or their call, texting has dropped then, you know, it should set off an alarm.

Clinician #2

Older adults perceived PGHD to be difficult to use as the volume of data would be too large, and it would be time-consuming to gain an understanding of their health and the effectiveness of care. Clinicians expressed the significance of a decision support system to act on the PGHD.

#### Theme 4: Perceived Clinical Benefits and Intrusiveness of PGHD

The monitoring aspect of PGHD disturbed the older adults to varying degrees. One older participant repeatedly expressed emotionally charged negativity toward PGHD collection and sharing of the data with clinicians. This was further perceived as a threat to autonomy. Clinicians acknowledged the tension between the clinical benefits and the intrusiveness of PGHD systems and felt that clinicians were accountable for gaining buy-in from patients:

It just seems to me very intrusive. Every little thing, every little step you take and so on...you get to a point where “I don’t want so much of you in my life.” I like the act that my doctor doesn’t overdo it. You thought about not wearing it and then you don’t get all the information.

Participant #2

And I guess I’m afraid I’m going to be told “You shouldn’t be doing this, you shouldn’t be doing this, you shouldn’t be doing that.” That’s hard to live with.

Participant #2

...gaining that buy-in and helping people understand that this data is going to help them in the long run.

Clinician #2

Clinicians also had heightened sensitivity to PGHD which might intrude patient privacy. One clinician perceived the monitoring of social media use for tracking mental health and GPS information for Alzheimer and dementia patients to be intrusive. The internal conflict between the clinical benefits and intrusiveness of PGHD was evident for one clinician:

Social media uses and communication felt a little intrusive...Yeah, the social media and the communication, I can see how that’s useful.

Clinician #2

When asked about the current regulations for patient privacy and confidentiality, clinicians viewed them as a necessary barrier and even as a facilitator for integrating PGHD into existing health information systems safely and securely:

...talking now between patient and provider, like that definitely needs to be given the most security that we can...so if you want to take information from a wearable device and throw it to an EMR or a hospital system, there’s sometimes a lot of challenges in being able to do that.

Clinician #2

the privacy laws are necessary...I would say it’s a, it’s definitely a barrier what between like healthcare provider sharing, So yeah, it is a, it’s a necessary barrier -

Clinician #3

### Perceived Usefulness of PGHD

When the frequency of the different types of PGHD mentioned in the focus groups was examined, it was noted that clinicians engaged in more diverse types of PGHD more frequently than older adults. Table 2 summarizes the PGHD asked in the Data Rating Questionnaire and the frequency of mention. Blood glucose level, step count, physical activity, sleep, and blood pressure were most frequently discussed.

**Table 2:** Frequency of patient-generated health data (PGHD) mentioned in focus group interviews.

PGHD	Frequency (how often was a concept mentioned?)		
	Clinicians (n=45), n (%)	Older adults (n=24), n (%)	Total (n-69), n (%)
Blood glucose	6 (13)	3 (13)	9 (13)
Step count	3 (7)	4 (17)	7 (10)
Physical activity	5 (11)	2 (8)	7 (10)
Sleep	3 (7)	4 (17)	7 (10)
Blood pressure	2 (4)	4 (17)	6 (9)
Gait	4 (9)	—^a^	4 (6)
Heart rate	2 (4)	2 (8)	4 (6)
Communication activity	3 (7)	—	3 (4)
Social media use	3 (7)	—	3 (4)
Stress level	—	3 (13)	3 (4)
Dietary intake	2 (4)	1 (4)	3 (4)
Body temperature	2 (4)	—	2 (3)
Body weight	1 (2)	1 (4)	2 (3)
GPS	1 (2)	—	1 (1)
Air quality	1 (2)	—	1 (1)
Ambient light	1 (2)	—	1 (1)
Air pressure	1 (2)	—	1 (1)
Body fat percentage	1 (2)	—	1 (1)
Mood	—	1 (4)	1 (1)
Typing pattern	1 (2)	—	1 (1)
Wound pictures	1 (2)	—	1 (1)
Sedentariness	—	—	—
EDA^b^	—	—	—
PEF^c^	—	—	—
Inhaler use	—	—	—

^a^Not mentioned.

^b^EDA: electrodermal activity.

^c^PEF: peak expiratory flow.

Stress level as PGHD was discussed only by older adults, and it was portrayed as having significant importance for overall well-being. Older adults also made a distinction between acute and chronic stresses:

If you have high stress and you have, what we would call a bad day, that affects your whole being, your whole body, and your mind more.

Participant #3

We get to this stage and many people have lost their spouse and it seems to take a really long—well, it never goes away. But to deal with stress is a high component.

Participant #2

**Figure 1:**
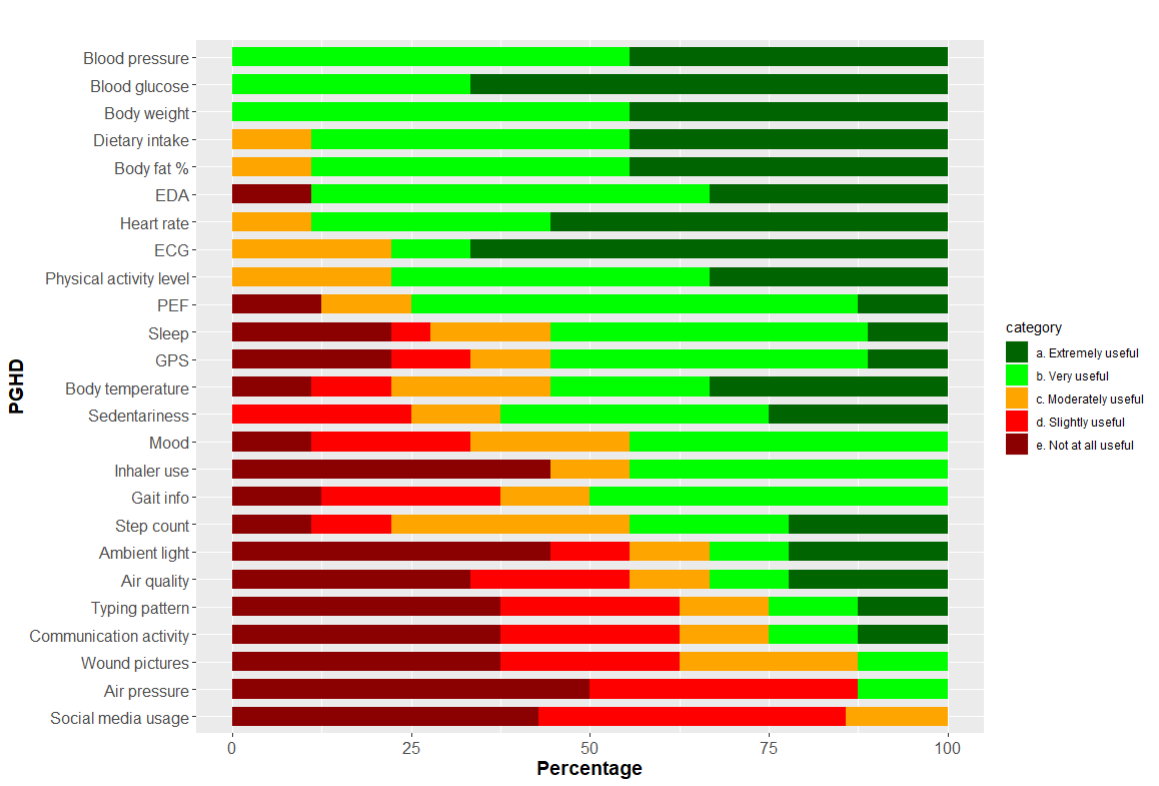
Bar graph showing the distribution of the Data Rating Questionnaire answers. ECG: electrocardiogram; EDA: electrodermal activity; PEF: peak expiratory flow; PGHD: patient-generated health data.

The Data Rating Questionnaire results showed that, on average, participants rated the usefulness of PGHD at 3.35, which is between moderately useful and very useful. The five most frequently mentioned types of PGHD (blood glucose, step count, physical activity, sleep, and blood pressure) had a higher average score of 3.83. The questionnaire results were significantly correlated with the frequency of PGHD mentioned in the focus group interviews (*r*=0.42; *P*=.03). Table 3 presents the average ratings of all PGHD for older adults and clinicians. Figure 1 shows the overall distribution of ratings for each PGHD type.

Clinicians tended to rate PGHD higher than older adults (mean 3.55 vs 3.18). The actively collected PGHD was rated significantly higher than the passively collected PGHD (mean 3.80 vs 3.05). Clinicians perceived passively collected PGHD as more trustworthy, as it prevented data manipulation by patients. However, the clinician ratings for actively and passively collected PGHD were similar.

**Table 3:** Average rating of patient-generated health data (PGHD) by older adults and cliniciansa.

PGHD			Rating (1=not at all useful and 5=extremely useful), mean (SD)				
			Older adults		Clinicians		Both older adults and clinicians
**Passively collected PGHD**							
	Step count	2.8 (1.10)		4 (1.41)		3.33 (1.32)	
	Gait	2.75 (0.96)		3.25 (1.50)		3.00 (1.20)	
	Physical activity	3.8 (0.45)		4.5 (1.00)		4.11 (0.78)	
	Sleep	2.9 (1.34)		3.38 (1.70)		4.44 (1.43)	
	Heart rate	4 (0.71)		5 (0.00)		3.63 (0.73)	
	Sedentariness	3.75 (1.26)		3.5 (1.29)		3.56 (1.19)	
	Body temperature	3.2 (1.64)		4 (1.15)		4.00 (1.42)	
	EDA^b^	3.6 (1.52)		4.5 (0.58)		3.11 (1.22)	
	GPS	2.8 (1.64)		3.5 (1.29)		2.67 (1.45)	
	Air quality	2.8 (1.64)		2.5 (1.91)		2.56 (1.66)	
	Ambient light	2.8 (1.79)		2.25 (1.89)		1.75 (1.74)	
	Air pressure	2 (1.41)		1.5 (0.58)		2.38 (1.66)	
	Communication activity	2.25 (1.50)		2.5 (1.73)		1.71 (1.51)	
	Social media use	1.67 (1.15)		1.75 (0.50)		2.38 (0.76)	
	Typing pattern	1.75 (0.96)		3 (1.83)		3.11 (1.51)	
**Actively collected PGHD**							
	Body weight	4.2 (0.45)		4.75 (0.50)		4.44 (0.53)	
	Body fat percentage	4.2 (0.45)		4.5 (1.00)		4.33 (0.71)	
	Blood glucose	4.4 (0.55)		5 (0.00)		4.67 (0.50)	
	Blood pressure	4.2 (0.45)		4.75 (0.50)		4.44 (0.53)	
	PEF^c^	3.75 (0.50)		3.5 (1.73)		3.63 (1.19)	
	Inhaler use	3.2 (1.30)		1.75 (1.50)		2.56 (1.51)	
	Wound pictures	2.25 (1.50)		2 (0.82)		2.13 (1.13)	
	ECG^d^	4 (1.00)		5 (0.00)		4.44 (0.88)	
	Mood	2.4 (1.14)		3.75 (0.50)		3.00 (1.12)	
	Dietary intake	4 (0.71)		4.75 (0.50)		4.33 (0.71)	

^a^Older adults: mean 3.18 (SD 0.82); clinicians: mean 3.56 (SD 1.12); both: mean 3.35 (SD 0.90).

^b^EDA: electrodermal activity.

^c^PEF: peak expiratory flow.

^d^ECG: electrocardiogram.

## Discussion

### Principal Findings

This study aimed to explore the perceptions of older adults and clinicians regarding PGHD and its perceived usefulness. The embedded mixed methods design allowed us to investigate the viewpoints of participants qualitatively and added specificity by quantitatively measuring the perceived usefulness of different types of PGHD. This approach augmented the findings from the focus group interviews with quantitative results by examining an additional aspect of PGHD while testing for the convergence of results from the two data sources.

Overall, we identified four major themes that older adults and clinicians perceived as influencing the use and sharing of PGHD. Participants perceived the objective nature of PGHD as asserting transparency in the patient-provider relationship. From the clinicians’ experience, patients tended to react negatively to the added transparency by stopping the collection of PGHD, selectively disclosing favorable data, and gaming the system. This view was reiterated by the patients. In general, people seek positive social interactions, and the patient-provider relationship is not an exception [23]. Patients display a natural tendency to *please the doctor*, and the older adults expressed fear and anxiety about the capacity of PGHD to highlight noncompliance with the care plan. As a result, it was perceived to have a negative impact on the patient-provider relationship. This finding expanded a recent interview study that called for the exploration of the unintended consequences of PGHD, which might include a feeling of failure or inadequacy on the part of health care consumers [24]. However, our findings directly contradicted those of previous studies [25]. Previously, PGHD was mainly viewed as a facilitator to enhance the patient-provider relationship with evidence for engaging patients in their care and increasing timely communication [26]. The difference in findings may be because the previous study focused on the effectiveness of PGHD from the perspective of system implementation and evaluation with limited insight into patient perception. In addition, our study sample showed contradicting views on their comfort level about disclosing noncompliant PGHD. This indicates the need for careful consideration of user preferences for data sharing and the need for flexibility in system design.

The accuracy, reliability, and validity of mHealth and wearable device-based PGHD have been previously identified as a common barrier for clinical use [15]. Our analyses identified poor reliability of data as a barrier, but the root cause for concern was the perceived lack of patient self-efficacy to carry out PGHD collection rather than the technical inaccuracies of the tools. Clinicians also voiced concerns about the perceived lack of understanding of the clinical relevance of PGHD collected by patients. Inadequate confidence in mHealth and wearable systems was identified where clinicians expressed the issue of inaccurate self-reported data. This theme highlighted the overall need for training and uncertainty about who is accountable for training the users. The need for patient training on collecting and recording PGHD has been a recurring theme in the literature [25]. Proper education may alleviate this issue, but the responsibility for educating patients is unclear when PGHD tracking is patient-initiated rather than clinician-initiated. Transferring the responsibility of educating patients about the proper use of PGHD systems to clinicians may not be an efficient use of resources as the lack of expertise in PGHD is a commonly reported barrier for clinicians [15]. This highlights the need for technical support for patients from health care organizations and recommends a higher standard for user-friendly interfaces for older adults.

Both clinicians and older adults discussed the uncertainty about the efficient ways of interpreting PGHD. Older adults had concerns about how the data are used by clinicians to benefit the care they receive. Clinicians voiced their lack of expertise in managing PGHD to extract relevant information. This was perceived as the main barrier for realizing the added clinical value of PGHD. As a result, a decision support system was viewed as an essential component of PGHD systems. This is in line with the recommendation that prioritizes a decision support system that can readily summarize PGHD and present the most relevant information as a key to integrating PGHD into electronic health records (EHRs) [27]. The need for a decision support system also extends to the patients’ use of PGHD. This can help them extract the most relevant and helpful information easily. However, only a handful of mHealth and wearable device systems have integrated decision support that can guide users to effectively turn information into meaningful actions [26,28]. Future studies should investigate the types of decision support that can be effectively delivered via mHealth.

Protecting patient privacy and confidentiality goes beyond complying with the minimum requirements imposed by regulations. Some older adults perceived the monitoring of PGHD as intrusive and perceived it as a threat to their autonomy. A similar sentiment was shared by clinicians, and sensitivity was particularly displayed toward GPS information, communication tracking, and social media use. Although concerned about its intrusiveness, clinicians saw the clinical benefits and the role of privacy regulations in enabling the collection of such information safely and securely. Furthermore, clinicians perceived that privacy regulations could facilitate the safe and secure integration of PGHD into health information systems. This view of the clinicians contradicts findings from the literature, indicating that many stakeholders view privacy concerns as a hindrance to the successful use of PGHD in clinical settings [15,16,25]. For example, patients were often unsure of privacy and confidentiality standards and regulations [25]. PGHD was sometimes shared with clinicians in noncompliant ways, further hindering its use by clinicians [16]. Privacy regulations are localized, and each jurisdiction faces unique challenges. Knowledge and expertise in health care exist for the integration of EHR systems, and parallels can be drawn with the integration of PGHD into EHR. Future studies should investigate possible solutions.

Older adults and clinicians tended to discuss the familiar types of PGHD, which were rated higher and as being more useful than other unfamiliar types of PGHD. The diversity of the PGHD discussed differed significantly. Clinicians ventured more frequently into discussions of PGHD types that were new to them than older adults and explored how they might add clinical value. This result was different from that of a previous study that tracked a range of PGHD collected by health care consumers and providers [24]. They found that health care consumers tracked a larger number of PGHD, and that clinicians focused on PGHD-related to their clinical specialty. The authors of this study did not share detailed information on the health care consumers, but we suspect that the difference may be due to differences in the study population. This was indicated when the most commonly tracked PGHD were wellness-focused, such as dietary intake, physical activity, and heart rate, whereas more clinical PGHD, such as blood pressure and blood glucose, were less frequently mentioned.

Clinicians carried out more extensive and detailed discussions on the clinical use of a range of PGHD than older adults. A significantly higher average PGHD rating by clinicians supports this finding. Clinicians indicated enhanced trustworthiness of passively collected data over actively collected data, as passive collection prevents patients from incorrect reporting. However, passively collected data were not rated as more useful by clinicians. This may be because the most highly rated PGHD, including blood glucose, blood pressure, body weight, and dietary intake, were actively collected. This represents a mismatch between state-of-the-art mHealth technology and the needs of patients and clinicians. Our participants explicitly mentioned that further advancement of sensor technology should lead to the expansion of passively collected data such as blood pressure and blood test results. This finding provides evidence for medical technology developers regarding clinicians’ data needs.

### Limitations

This study had several limitations. The small number of participants in the focus group interviews limited the concepts from reaching saturation. This limitation was partly alleviated as more than 80% of all themes are usually discovered within two to three focus group sessions [29], and partly through the collection of quantitative data to augment the qualitative results. Only young clinicians were interested in participating in the study. Owing to this convenience sampling, the absence of older clinicians is a limitation of this study. The composition of the focus group sessions, comprising older adults and clinicians, was uneven. This may have influenced the dynamics of the discussions to be narrower in scope, as one group of participants may have not been able to express their opinions freely. Even within each group, participants were likely to simply confirm other participants’ opinions (ie, confirmation bias). The analyses of the study results were conducted by a single reviewer, which may have introduced bias and personal views in the coding process and theme synthesis. Our older adult participants were members of a research support group, and as a result, there may have been a representative bias. Limited information about the topic was provided before the focus group, and some participants were unfamiliar with the topic of PGHD. Although the lack of understanding of PGHD may have limited the breadth and depth of discussion, this was done intentionally to capture the true perceptions of older adults and clinicians. Finally, the Data Rating Questionnaire was not piloted before the study.

### Conclusions

This embedded mixed methods study generated several important findings about older adult and clinician perceptions and perceived usefulness of a range of PGHD. The increasing popularity and adoption of consumer wearable devices and mHealth apps, especially among older adults, will continue to lead to an increasing demand for better integration of PGHD into health care systems. The volume and complexity of PGHD will also continue to increase with the advancement of sensor technologies, and the borderline between consumer and medical devices has already started to blur. PGHD presents new opportunities to improve the care clinicians provide and increase the efficiency of the health care system. Such momentous opportunities have been recognized by governments around the world, and foundational work has begun in many countries. Nevertheless, there is a need for more evidence to identify obstacles for health care users, providers, organizations, and decision makers. Greater insight into these barriers can inform users, providers, developers, and other stakeholders of the priorities for the effective integration of PGHD into health care. In particular, concerns about data privacy, potential negative impact on the trust between older adults and clinicians, data quality and quantity, and usability of PGHD-related technologies will need to be investigated and addressed further.

## Appendix

Multimedia Appendix 1 Data rating questionnaire.

Multimedia Appendix 2 Group discussion guide.

Multimedia Appendix 3 A case study of a 77-year-old man newly diagnosed with congestive heart failure with pre-existing type 2 diabetes, hypertension, and hyperlipidemia.

## Abbreviations

EHR: electronic health record
EMA: ecological momentary assessment
mHealth: mobile health
PGHD: patient-generated health data
PROM: patient-reported outcome measure
