# Supplementary material for: Clinicians and Older Adults’ Perceptions of the Utility of Patient-Generated Health Data in Caring for Older Adults: Exploratory Mixed Methods Study
Source: JMIR Aging. 2021 Nov 5;4(4):e29788. doi: 10.2196/29788 (PMC8663681; doi:10.2196/29788)
Supplement: Multimedia Appendix 1 [file aging_v4i4e29788_app1.docx]

## Multimedia Appendix 2. Data Rating Questionnaire

**Data Rating Questionnaire**

Please rate the following personal sensing data based on your opinion on their usefulness to the case scenario.

| Passively-sensed data: Data that are collected without user input. Minimal effort. | | | | | |
| --- | --- | --- | --- | --- | --- |
|  |  |  |  |  |  |
| Data Type | **Not at all useful** | **Slightly useful** | **Moderately useful** | **Very useful** | **Extremely useful** |
|  |  |  |  |  |  |
| Step count |  |  |  |  |  |
|  |  |  |  |  |  |
| Gait information (e.g. stride) |  |  |  |  |  |
|  |  |  |  |  |  |
| Physical activity level (e.g., duration, frequency, intensity) |  |  |  |  |  |
|  |  |  |  |  |  |
| Sleep quantity (e.g. total sleep time, time took to fall asleep) |  |  |  |  |  |
|  |  |  |  |  |  |
| Sleep quality (e.g. sleep efficiency, deep sleep and light sleep, nighttime awakenings) |  |  |  |  |  |
|  |  |  |  |  |  |
| Heart rate (e.g. average heart rate, resting heart rate) |  |  |  |  |  |
|  |  |  |  |  |  |
| Sedentariness level (i.e. duration, frequency) |  |  |  |  |  |
|  |  |  |  |  |  |
| Body temperature |  |  |  |  |  |
|  |  |  |  |  |  |
| Electrodermal activity (measures stress level and emotional arousal) |  |  |  |  |  |
|  |  |  |  |  |  |
| GPS/Location |  |  |  |  |  |
|  |  |  |  |  |  |
| Air quality |  |  |  |  |  |
|  |  |  |  |  |  |
| Ambient light |  |  |  |  |  |
|  |  |  |  |  |  |
| Barometer (i.e. air pressure) |  |  |  |  |  |
|  |  |  |  |  |  |
| Communication activities (i.e. call, texting) |  |  |  |  |  |
|  |  |  |  |  |  |
| Social media uses (i.e. Facebook, Twitter) |  |  |  |  |  |
|  |  |  |  |  |  |
| Typing patterns (i.e. text linguistic, speed, accuracy) |  |  |  |  |  |
|  |  |  |  |  |  |
|  |  |  |  |  |  |
|  |  |  |  |  |  |
|  |  |  |  |  |  |
|  |  |  |  |  |  |
| Actively-sensed data: Data that are collected with user input. More effort needed. | | | | | |
| Data Type | **Not at all useful** | **Slightly useful** | **Moderately useful** | **Very useful** | **Extremely useful** |
|  |  |  |  |  |  |
| Weight |  |  |  |  |  |
|  |  |  |  |  |  |
| Body fat % |  |  |  |  |  |
|  |  |  |  |  |  |
| Blood glucose level |  |  |  |  |  |
|  |  |  |  |  |  |
| Blood pressure |  |  |  |  |  |
|  |  |  |  |  |  |
| Peak expiratory flow |  |  |  |  |  |
|  |  |  |  |  |  |
| Inhaler usage (i.e. puffer for asthma) |  |  |  |  |  |
|  |  |  |  |  |  |
| Pictures (i.e. skin cancer, wound) |  |  |  |  |  |
|  |  |  |  |  |  |
| Electrocardiography (i.e. smartphone paired ECG device) |  |  |  |  |  |
|  |  |  |  |  |  |
| Mood (i.e Mood journal) |  |  |  |  |  |
|  |  |  |  |  |  |
| Dietary intake (i.e Food journal) |  |  |  |  |  |
| Other data types: What other data you may find useful? Tell us what they are. | | | | | |
|  | | | | | |
| Data Type | **Not at all useful** | **Slightly useful** | **Moderately useful** | **Very useful** | **Extremely useful** |
|  |  |  |  |  |  |
|  |  |  |  |  |  |
|  |  |  |  |  |  |
|  |  |  |  |  |  |
