# Supplementary material for: Clinicians and Older Adults’ Perceptions of the Utility of Patient-Generated Health Data in Caring for Older Adults: Exploratory Mixed Methods Study
Source: JMIR Aging. 2021 Nov 5;4(4):e29788. doi: 10.2196/29788 (PMC8663681; doi:10.2196/29788)
Supplement: Multimedia Appendix 2 [file aging_v4i4e29788_app2.docx]

## Multimedia Appendix 3. Group Discussion Guide

**Group Discussion Guide (30 – 40 minutes)**

**Disclosure of audio recording**

- We have spent the last 20 – 30 minutes completing the data rating activity where you rated different types of data that can be collected from mobile devices that we use daily such as smartphones and wearable devices. Over the next half an hour we will discuss as a group some of the ratings you have given to different data types.
- Before we jump into the next session where we will have a group discussion about personal sensing, I would like to assure you that the study has been reviewed and received ethics clearance through the University of Waterloo Office of Research Ethics.
- If you feel that you are uncomfortable speaking about a specific subject, you have the right to refuse any questions or topics you may not want to discuss.
- This discussion session will be audio-recorded for the purpose of data analysis. This audio-recording will not be published nor used for any other purpose. Excerpts from the recording may be included in the thesis and/or publications resulting from this research. All excerpts will be made anonymous with no personal identifying information.

**Introduction to the group discussion session**

- The goal of this discussion is not to find a consensus or reach an agreement, but rather share your thoughts and listen to others to understand how individuals with different backgrounds view things similarly or differently.
- We will discuss some of the highly and lowly rated data types reflecting the two case scenarios. I am here as a facilitator to keep track of time and to guide the discussion. I will limit my participation in the discussion itself.

**Areas of interest to discuss and explore:**

- Reflecting back to the Case Study 1 (5-10 minutes):
  - What data types did you rate the highest/extremely useful?
  - Why did you rate them high/extremely useful?
    1. Probes/Prompts
       1. Can you tell us specific aspects of Mr. McDonald’s life that these data played a key role?
       2. How were the data used to help Mr. McDonald?
          - What specific challenge(s) did the data help resolve?
       3. How do these data help clinicians that Mr. McDonald interacts with (i.e., pharmacist, care coordinator, family physician)?
       4. How do these data help caregivers (i.e., family members, friends)?
  - What are the data types that you rated low/least useful?
    1. Probes/Prompts
       1. Can you tell us what made it less useful?
          - Think about technical, social, organizational and other aspects
       2. Can you think of ways to increase the usefulness of these data?
          - Think about technical, social, organizational and other aspects
- Reflecting back to the Case Study 2 (5-10 minutes):
  - What data types did you rate the highest/extremely useful?
  - Why did you rate them high/extremely useful?
    1. Probes/Prompts
       1. Can you tell us specific aspects of Mrs. Offnfall’s life that these data played a key role?
       2. How were the data used to help Mrs. Offnfall?
          - What specific challenge(s) did the data help resolve?
       3. How do these data help clinicians that Mrs. Offnfall interact with (i.e. physiotherapist, family physician)?
       4. How do these data help caregivers (i.e. family members, friends)?
  - What are the data types that you rated low/least useful?
    1. Probes/Prompts
       1. Can you tell us what made it less useful?
          - Think about technical, social, organizational and other aspects
       2. Can you think of ways to increase the usefulness of these data?
          - Think about technical, social, organizational and other aspects
- Prompts for thinking outside the case scenarios (5-10 minutes)
  - Provided case scenarios focused on specific health issues. Based on your experience with and knowledge about current healthcare system:
    1. Can you think of types of data we talked about today that are used in healthcare currently?
    2. What are the types of data that you have rated highly but are not used?
    3. Can you think of any reasons why they are not used?
    4. Other prominent examples:
       1. Hypertension management, pregnancy monitoring, atrial fibrillation monitoring, physical activity monitoring, sleep monitoring, diabetes management, skin cancer diagnostic
- Key aspects that can be probed further throughout the discussion:
  - Technical aspect
    1. Integration with the existing system
    2. Validity and reliability of technology
  - Social aspect
    1. Clinician vs patient-initiated data collection
  - Clinical aspect
    1. Clinician competency in interpreting data
    2. Doctor-patient relationship and communication tool
    3. Patient safety
  - Organizational aspect
    1. Clinical workflow
    2. Time commitment
  - Privacy and confidentiality

**Finish**

- Is there anything else you would like to add to the discussion or talk to me about?
- Thank you for time and effort.
