# Supplementary material for: Clinicians and Older Adults’ Perceptions of the Utility of Patient-Generated Health Data in Caring for Older Adults: Exploratory Mixed Methods Study
Source: JMIR Aging. 2021 Nov 5;4(4):e29788. doi: 10.2196/29788 (PMC8663681; doi:10.2196/29788)
Supplement: Multimedia Appendix 3 [file aging_v4i4e29788_app3.docx]

## Multimedia Appendix 1. Case study

**Case Study 1**

**Mr. Greg McDonald**, 77-year-old man, was diagnosed with congestive heart failure a year ago after experiencing chest pain following a big family dinner. His multiple medical conditions include type 2 diabetes, high blood pressure, and high cholesterol.

Before being discharged from the hospital, Mr. MacDonald was counselled on a home diuretic protocol by a care coordinator. Mr. McDonald was given a logbook and asked to measure his weight, blood pressure, heart rate, blood oxygen level, and body temperature twice a day, once in the morning and again at night. He was also asked to answer questions about heart failure symptoms daily. The first few pages of the logbook are Mr. McDonald’s personalized treatment plan as filled out and explained by his cardiologist and the care coordinator on site. It instructs on when to increase the dose of diuretic drugs and take additional drugs.

Mr. McDonald was also referred to a pharmacist (on site) for pharmacotherapy assessment and diabetes management. His diabetes is currently being treated with a fast-acting insulin. Mr. McDonald occasionally “takes a little more” insulin when he notes high blood sugar readings. The pharmacist changed to a slow-acting insulin and he was explained the dosing concept on the new medications and how this regimen can give him greater flexibility.

The care coordinator signed out an iPad and glucometer and showed how to use the patient portal app to record the measurements. Through the app, he can access a sliding scale to correct for any temporary elevation of blood glucose. He was told to test four times daily and to record his blood glucose results, carbohydrate intake, and insulin doses in the app. Mr. McDonald was told that these data are accessible by the pharmacist who will adjust the insulin based on the recordings. Mr. McDonald was advised to pick up the adjusted doses when the pharmacist contacts him and continue with the iPad.

Mr. McDonald was discharged and made his way home with the help of his family. With him, he has a stack of information brochure, a logbook, and a pulse oximeter for his new heart condition, along with an iPad and glucometer for his diabetes.
